# Efficacy of prostaglandin E2 versus prostaglandin F2 alpha assisted with narrowband-UVB in stable vitiligo

**DOI:** 10.1007/s00403-023-02700-8

**Published:** 2023-08-18

**Authors:** Yomna Mazid El-Hamd Neinaa, Myada Abd Elmohsen Mahmoud, Gamal Mohamed El Maghraby, Zeinab Abd Elsamd Ibrahim

**Affiliations:** 1 Faculty of Medicine, Tanta University, Tanta, Egypt; 2 Zifta General Hospital, Zifta, Elgharbia Egypt; 3 Faculty of Pharmacy, Tanta University, Tanta, Egypt

**Keywords:** Vitiligo, PGF2α, PGE2, NB-UVB

## Abstract

In the recent decades, prostaglandins were recommended as a new therapeutic modality of stable vitiligo with promising efficacy. Therefore, we designed the current work to compare the significance of two different subtypes of prostaglandins [prostaglandin E2 (PGE2) versus prostaglandin F2 alpha (PGF2α)], assisted with NB-UVB phototherapy, in treatment of stable vitiligo. This study was conducted on 30 patients with stable non-segmental vitiligo. Three approximately similar vitiliginous areas were chosen in each patient and assigned into 3 groups. Each group treated with intradermal injection of either PGE2 (group I), PGF2α (group II), or saline as placebo (group III) at frequency once/week for 12 weeks. Concomitantly, all groups received NB-UVB phototherapy twice weekly for 3 months. The outcomes of this study discovered that the therapeutic efficacy of intradermal injection of either PGE2 or PGF2α assisted with NB-UVB phototherapy was comparable with non-significant difference between them in spite of being significantly higher than NB-UVB alone. However, there were a significantly earlier onset of repigmentation and higher degree of satisfaction regarding areas treated with PGE2 than those treated with PGF2α. In conclusion, both PGF2α and PGE2 intradermal injection could be considered as quite simple and affordable techniques in the treatment of stable vitiligo with no reported side effects and good patient satisfaction.

## Introduction

Skin depigmented patches of vitiligo create a significant deleterious effect on the patients' emotional and social interactions, even if they do not reduce life expectancy. Several hypotheses were suggested for loss of melanocytes described in vitiligo, including autoimmune, genetic, neurological, and biochemical mechanisms [1]. Oxidative stress were also suggested to participate in melanocyte destruction, through inhibition of glutathione with subsequent suppression of prostaglandin isomerase enzyme resulting in prostaglandin E2 (PGE2) deficiency [2].

Prostaglandin E2 (PGE2) is endogenously synthesized in the skin and reported to play an active role in immunomodulation, melanogenesis, and maturation of melanocytes [3]. Hence, it was formerly recommended as topical treatment of vitiligo and reported successful results [2, 3].

Prostaglandin F2 alpha (PGF2α) is another type of prostaglandins that is commercially available as pharmaceutical preparation known as latanoprost eye drops for reduction of high intraocular pressure [4]. Based on the observations that glaucoma patients developed peri-ocular and iridal hyperpigmentation as adverse outcomes of the use of PGF2α, it was studied for its therapeutic value in vitiligo [5]. PGF2α was reported to be an effective vitiligo treatment either topical [6], transdermal with microneedling [7] or intradermal injection [8], with enhanced efficacy when combined with phototherapy [6].

None of the previous studies evaluated PGE2 use through intradermal route or assisted with NB-UVB in treatment of vitiligo. Therefore, we tried to evaluate the significance of intradermal injection of PGE2, assisted with NB-UVB phototherapy in treatment of vitiligo and to compare it with PGF2α.

## Patients and methods

This is a randomized double blinded prospective comparative controlled study that was conducted at vitiligo unite in dermatology department of Tanta University Hospitals—Egypt, in the period from June 2021 to June 2022 after approval by Institutional Review Broad (IRB), Code No: 34248/11/20, and per the declaration of Helsinki. This study included 30 patients with stable non-segmental vitiligo (VIDA score ≤ 1) having at least 3 approximately similar vitiliginous areas and acknowledged to contribute into the study and signed informed consent. Patients with any other dermatologic, systemic or immunologic disorder, and those who used any topical or systemic treatment for vitiligo during the preceding 3 months were excluded.

### Randomization

In each patient, 3 approximately similar vitiliginous areas (regarding size, site, and duration) with surface area less than 20 cm^2^ were chosen in each patient, and divided randomly into 3 groups, each of which received intradermal injection session at frequency once per week for 3 months as follows:

- Group I treated by intradermal injection of PGE2
- Group II treated by intradermal injection of PGF2α
- Group III received intradermal injection of saline (as placebo)

In addition, all patients received concomitant NB-UVB twice weekly for 3 months and followed up for another 3 months.

### Prostaglandins preparation

Unfortunately, neither PGE2 nor PGF2a is available in Egypt as injection formula. PGE2 ‏is commercially available as dinoprostone vaginal tablets 3 mg, (Prostin E2, Pfizer, Sanico NV, Turnhout, Belgium). To prepare solution suitable for intradermal injection, each tablet was transformed into powder in a separate vial, and then disinfected by Cobalt-60 gamma radiation. During treatment session, each vial was dissolved with 12 ml saline; each 1 mL of it contains 250 μg of dinoprostone. It was stored at 2–8 °C for a maximum of 2 weeks. PGF2α is commercially available as Latanoprost 0.005% (Xalatan eye-drop formulation 2.5ml; Pfizer Manufacturing, Puurs, Belgium). Each 1 mL of Xalatan contains 50 μg of latanoprost. It was stored at 2–8 °C. According to Eldelee et al. [8], PGF2α eye-drop was injected intradermally as such without any dilution. Once opened, the container was stored at room temperature below 25 °C away from direct light, for a maximum of 4 weeks.

### Intradermal injection sessions

Topical anesthetic cream was applied for 20–30 min to the chosen vitiliginous areas (each ≤ 20 cm^2^) and then sterilized by 70% alcohol immediately before each injection session. 1 mL insulin syringes (28G × ½) were used for intradermal injection at depth of about 3.5 mm, and 1 cm distance was kept between each injection point. 1–2 mL prostaglandin was injected intradermally every session (0.1 mL/injection) with a maximum dose of 500μg PGE2/session and 100μg PGF2α/session. This approach was repeated weekly in all patients until improvement or for a maximum of 12 sessions (3 months).

### Phototherapy sessions

Every patient obtained twice-weekly NB-UVB treatment sessions for a maximum of 3 months, or until complete repigmentation was achieved. Eight NB fluorescent bulbs with a spectrum of 310–315 nm and a maximum wave length of 311 nm, mounted in a Waldmann UV-100 unit, served as the NB-UVB source (Philips TL 100, Hamburg, Germany). The UVB exposure was adjusted as 0.33 J/cm^2^ and then was subsequently increased by 20% per session until the minimal erythema dose was reached. The chosen vitiliginous areas were exposed while both eyes were protected by UV-blocking eyewear during the NB-UVB sessions.

### Evaluation of the treatment outcomes

Qualitative evaluation of the therapeutic effects was done by a three-physicians committee at the end of the study (3 months following last treatment session) by comparing before and after digital photographs. The repigmentation responses were expressed according to the Visual Analog System Score (VAS) [9] as follows: grade I (poor) if percent of repigmentation was less than 25%, grade II (fair) 25–50%, grade III (good) 50–75%, grade IV (excellent) if percent of repigmentation was more than 75%.

### Patients’ satisfaction

The patients were finally requested to express the degree of their satisfaction with the treatment outcomes as follows: not satisfied, slightly satisfied, satisfied, or very satisfied.

### Adverse reactions

The treated vitiliginous areas were carefully assessed for any adverse reactions developed throughout the study.

### Statistical analysis

The patients’ data were studied using the SPSS statistical software version 20.0. The employed tests were labeled beneath the tables. Significance of the gained results was refereed at the 5% level.

## Results

The patients’ age ranged from 10 to 65 years with a mean of 34.17 ± 17.10. Females were predominant and constituted 73.3% of the studied patients (22 patients). The disease duration ranged from 0.67 to 35 years with a mean of 8.88 ± 8.94. Twelve patients (40%) had phototype III skin and 18 patients (60%) had phototype IV skin.

**Fig. 1:**
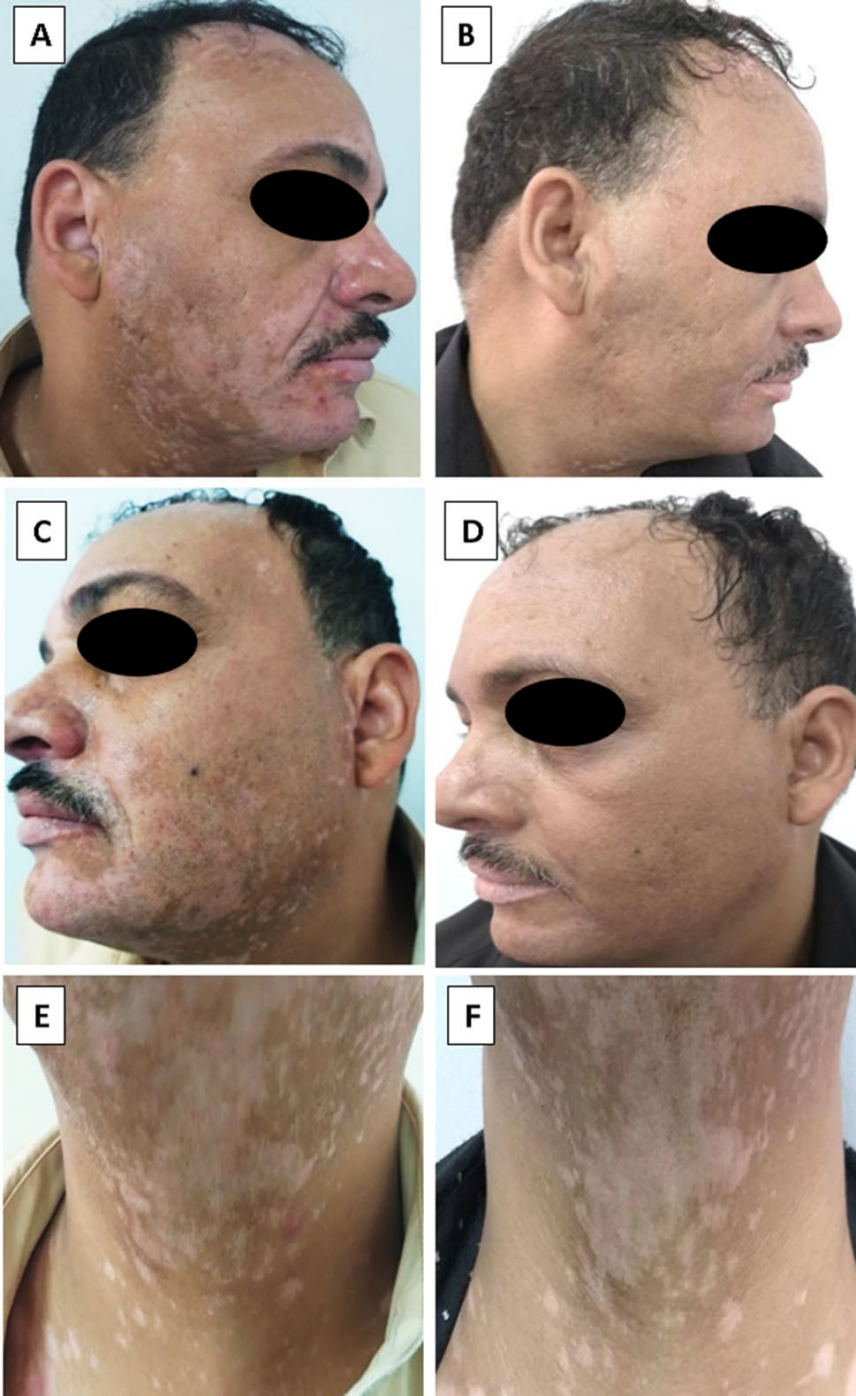
Vitiligo on face and neck. **a**, **b** pre- and post-treatment with intradermal injection of PGE2 assisted with NB-UVB reporting grade IV (excellent) improvement, **c**, **d** pre- and post-treatment with intradermal injection of PGF2α assisted with NB-UVB reporting grade IV (excellent) improvement, **e**, **f** pre- and post-treatment with NB-UVB alone reporting grade I (poor) improvement

**Fig. 2:**
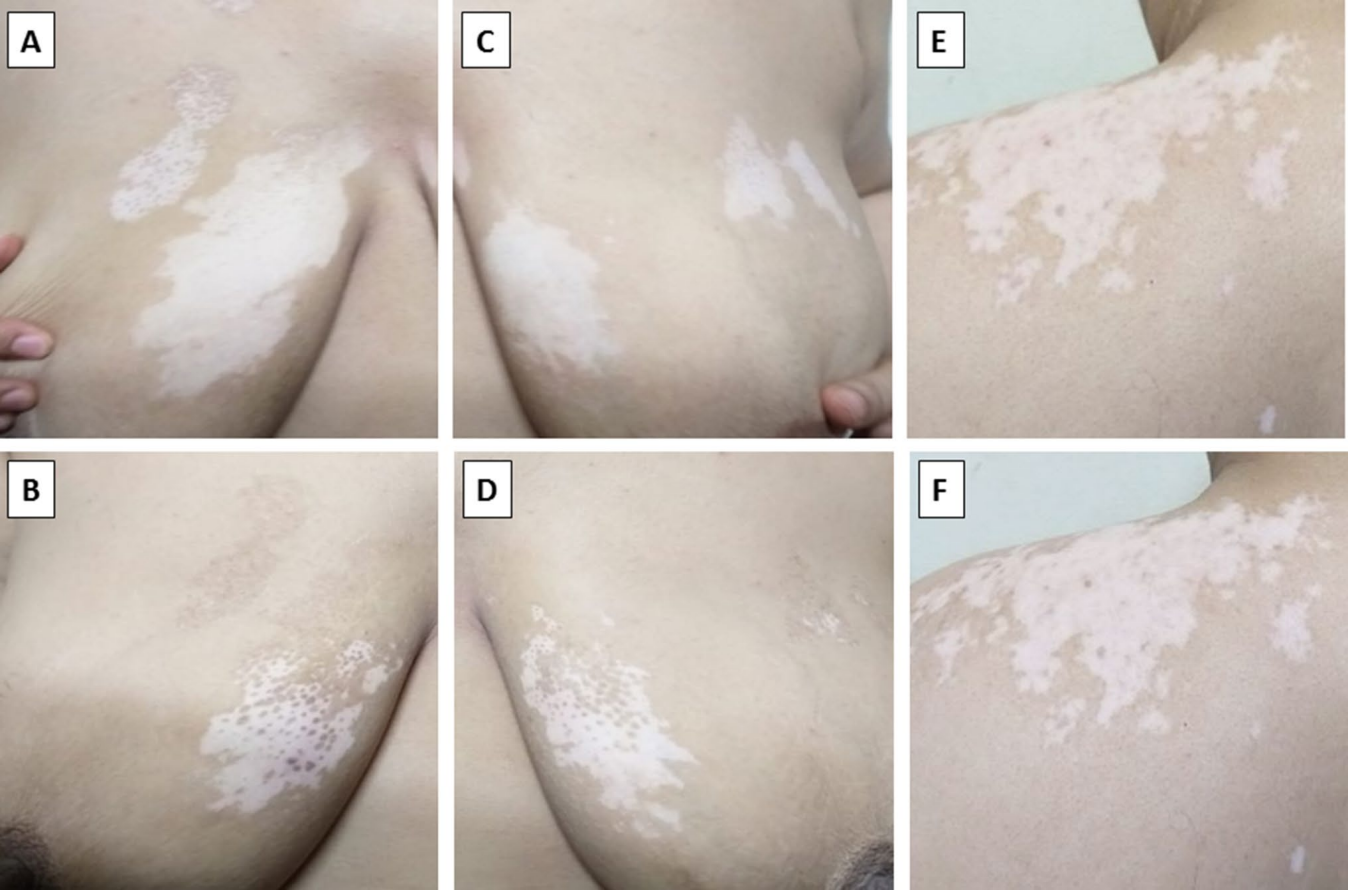
Vitiligo on trunk **a**, **b** pre- and post-treatment with intradermal injection of PGE2 assisted with NB-UVB reporting grade III (good) improvement, **c**, **d** pre- and post-treatment with intradermal injection of PGF2α assisted with NB-UVB reporting grade III (good) improvement, **e**, **f** pre- and post-treatment with NB-UVB alone reporting no improvement

Variable degrees of clinical improvement were reported in all studied groups, with the highest degree noticed in group I (93.3% of chosen vitiliginous areas) followed by group II (90% of chosen vitiliginous areas) but with non-significant difference between them (p1 = 0.956). The least degree of clinical improvement was reported in group III (66.7% of chosen vitiliginous areas). Although excellent improvement was evident in 9 vitiliginous areas (30%) treated by PGE2 (group I), and 8 vitiliginous areas (26.7%) treated by PGF2α (group II), none of group III treated areas reported excellent improvement. VAS score among group I and II vitiliginous areas treated by NB-UVB assisted by both types of prostaglandins showed significantly higher degree of clinical improvement than those treated by NB-UVB only (group III) with *p*2 < 0.001*, *p*3 = 0.001* respectively, (Figs. 1, 2, Table 1).

**Table 1:** Comparison between the three studied groups in vitiligo patients

	Group I (PGE2 + NB-UVB)	Group II (PGF2α + NB-UVB)	Group III (NB-UVB only)	Test of Sig.	*p*
Onset of repigmentation					
Min.–max	3.0–10.0	3.0–11.0	6.0–12.0	*F* = 19.073*	< 0.001*
Mean ± SD	5.61 ± 2.20	7.33 ± 2.27	9.40 ± 1.67		
Median (IQR)	5.50 (3.50–7.0)	8.0 (6.0–9.0)	9.50 (8.0–11.0)		
	*p*_1_ = 0.009*, *p*_2_ < 0.001*, *p*_3_ = 0.004*				
Percent of repigmentation					
Min.–Max	0.0–100.0	0.0–100.0	0.0–60.0	*H* = 21.805*	< 0.001*
Mean ± SD	54.40 ± 31.46	48.67 ± 33.01	18.70 ± 18.66		
Median (IQR)	60.0 (30.0–80.0)	52.50 (20.0–75.0)	15.50 (0.0–25.0)		
	*p*_1_ = 0.492, *p*_2_ < 0.001*, *p*_3_ < 0.001*				
Visual analog system score (VAS)					
Grade I (poor)	7 (23.3%)	10 (33.3%)	23 (76.7%)	*χ*^2^ = 22.172*	0.001*
Grade II (fair)	6 (20.0%)	5 (16.7%)	4 (13.3%)		
Grade III (good)	8 (26.7%)	7 (23.3%)	3 (10.0%)		
Grade IV (excellent)	9 (30.0%)	8 (26.7%)	0 (0.0%)		
	*p*_1_ = 0.862, ^MC^*p*_2_ < 0.001*, ^MC^*p*_3_ = 0.001*				
Patients satisfaction					
Not satisfied	2 (6.7%)	3 (10.0%)	17 (56.7%)	*χ*^2^ = 42.195*	^MC^*p* < 0.001*
Slightly satisfied	6 (20.0%)	16 (53.3%)	6 (20.0%)		
Satisfied	4 (13.3%)	2 (6.7%)	6 (20.0%)		
Very satisfied	18 (60.0%)	9 (30.0%)	1 (3.3%)		
	^MC^*p*_1_ = 0.032*, *p*_2_ < 0.001*, *p*_3_ < 0.001*				

*H*: *H* for Kruskal–Wallis test, pairwise comparison bet. each 2 groups was done using post hoc test (Dunn’s for multiple comparisons test)

*F*: *F* for one way ANOVA test, pairwise comparison bet. each 2 groups was done using post hoc test (Tukey)

*p*: *p* value for comparing between the studied groups

*p*_1_: *p* value for comparing between group I and group II

*p*_2_: *p* value for comparing between group I and group III

*p*_3_: *p* value for comparing between group II and group III

*IQR* inter quartile range, *SD* standard deviation, *χ*^*2*^ Chi square test, *MC* Monte Carlo

*Statistically significant at *p* ≤ 0.05

The onset of repigmentation initiated after a mean of 5.61 ± 2.20 sessions in group I, 7.33 ± 2.27 sessions in group II, and 9.40 ± 1.67 sessions in group III with statistically significant difference among the three groups (*p*1 = 0.009*, *p*2 < 0.001*, *p*3 = 0.004*) (Table 1).

There was statistically significant negative correlation regarding the degree of improvement evaluated by repigmentation percent and disease chronicity among all studies groups as follows: group I (*r* = − 0.363, *p* = 0.049*), group II (*r* = − 0.524, *p* = 0.003*), and group III (*r* = − 0.470, *p* = 0.009*).

The studied patients were significantly satisfied with the therapeutic outcomes of PGE2 more than PGF2α with ^MC^*p*1 = 0.032*. Obviously, higher degrees of satisfaction were stated with the therapeutic outcomes of intradermal injection of both types of prostaglandins analogs (PGE2 and PGF2α) assisted with NB-UVB than NB-UVB phototherapy only with *p*2 and *p*3 < 0.001* (Table 1).

Temporary insignificant pain, erythema and mild burning sensation were the main complains reported among the enrolled patients. Disease activation or deterioration not observed in any patient. No recurrence reported in any group till the end of the study.

## Discussion

Cure of vitiligo is quiet challenging and the majority of therapeutic approaches rely on those targeting inflammatory and immune responses, like topical or systemic steroids or topical calcineurin inhibitors, which are frequently used in combination with phototherapy to encourage melanogenesis [10]. In the last decades, prostaglandins were recommended for vitiligo, and two types of them (PGF2α and PGE2) were investigated for their therapeutic efficacy in promoting repigmentation [2, 3, 6, 11]. PGE2 was the first type of prostaglandins studied for its efficacy as topical treatment of vitiligo and reported appreciated results [2, 3]. Later on, PGF2α was considered and reported great success [6–8]. To date, no previous published work compared their significance in inducing repigmentation of vitiligo lesions.

In the present study, PGE2 treated vitiliginous areas reported variable degrees of clinical improvement in 93.3% of all studied patients, and just 6.7% showed no repigmentation at all. Promisingly, good to complete repigmentation was reported in 56.67% of them. Our findings were virtually comparable to that of Parsad et al. [3] who applied topical PGE2 gel (166.6 µg/g) on the depigmented skin of vitiligo patients once daily for 6 months. In addition, Kapoor et al. [2] reported approximately similar findings on using PGE2 gel twice daily for 6 months, on a larger group of vitiligo patients. To our knowledge, this study is the first one that evaluated PGE2 through intradermal route, to improve the outcome of NB-UVB in vitiligo treatment.

Regarding PGF2α treated vitiliginous areas, variable degrees of clinical improvement were noticed in 90% of the enrolled patients, and 50% of them reported good to complete repigmentation. The current work outcomes were nearly comparable to Eldelee et al. [8], favoring their study. The smaller size of the vitiliginous patches they treated and the locations of the patients' diseases may clarify this slight discrepancy.

The successful treatment outcomes of PGF2α in vitiligo were formerly described when used topically ‏[6]. After that, topical PGF2α was proved to be more effective when combined with phototherapy‏ [6] or both microneedling and phototherapy‏ [7]. However, the best therapeutic outcomes were reported to be achieved when topical PGF2α used concomitantly with laser therapy (Fraxel Erbium) and then UVA-1 laser ‏ [12], and more recently when used as intradermal injection‏ [8]. The therapeutic success of prostaglandins in provoking repigmentation of vitiliginous areas were suspected to be the result of activation of tyrosinase enzyme with subsequent enhancement of melanin production, in addition to activation of melanocytes multiplication‏ [13]. Furthermore, prostaglandins have immunomodulatory effects participating in turning off the autoimmune destruction of melanocytes which is the hallmark of vitiligo‏ [3]. In contrary to our results, Nagui et al. [14] recommended anti-PGF2a drugs for vitiligo treatment based on their detection of high levels of PGF2α in vitiliginous and non-vitiliginous skin of their studied patients when compared to healthy controls, and therefore, they suggested a possible role of PGF2α as an important marker of oxidative stress in vitiligo pathophysiology. They proposed that repigmentation of vitiliginous lesions reported in all previous studies [6–8, 12] that evaluated the efficacy of PGF2α in vitiligo may be attributed to the base substance and not the active ingredient in a mechanism similar to that inducing Berloque dermatitis [14].

In the current study, by evaluating the therapeutic outcomes of phototherapy assisted with intradermal injection of PGE2 in comparison to that of PGF2α, non-significant difference was described between them in spite of slight higher efficacy of PGE2. This finding could be attributed to the direct specific stimulatory effect of PGE2 on melanocytes versus indirect nonspecific effect of PGF2α as proposed by Nagui et al. [14]. On the other hand, phototherapy assisted with either PGE2 or PGF2α reported to be more significantly effective than phototherapy alone. Numerous factors could account for the notable improvement in lesions treated with prostaglandins (whether PGE2 or PGF2α) and NB-UVB together as opposed to those treated with NB-UVB alone. It had been suggested that NB-UVB activates endogenous prostaglandins that works synergistically with that injected intradermally [15]. Additionally, collaboration was suggested between injected prostaglandins and further mediators generated in response to NB-UVB phototherapy, such as endothelin-1, alpha-melanocyte-stimulating hormone, adrenocorticotrophic hormone, stem cell factor, and nerve growth factor [16]. Moreover, NB-UVB phototherapy increases the expression of prostaglandin FP receptors, which enhances the effects of intradermally injected prostaglandins [17].

Interestingly, significant earlier onset of repigmentation with subsequent higher degrees of patient satisfactions were noticed in PGE2 treated vitiliginous areas than those treated by PGF2α. This could be explained by the probability that the intradermal injection of exogenous PGE2 activates melanocytes directly with subsequent rapid induction of repigmentation. On the other hand, PGF2α exerts its effect indirectly through stimulation of endogenous cyclooxygenase (COX-2) that act as a mitogenic and inflammatory stimulus activating endogenous PGE2 in keratinocytes [6, 18] with subsequent slight delay in its effect. Interestingly, the cumulative therapeutic effects of both types of prostaglandins were relatively matching at the end of the study with non-significant difference between them.

In accordance with most of the previous studies [2, 3, 6], inverse correlation was detected between the therapeutic outcomes of the 3 treatment modalities tried in the current work and disease chronicity that mostly referred to loss of follicular melanocytes reservoir in persistent long-term depigmented lesions [19–21].

Limitations of the present study include small sample size, choosing vitiliginous areas located in various body sites, use of different dosage forms of both PGE2 and PGF2α in addition to short-term follow-up period.

In conclusion, the intradermal injection of either PGE2 or PGF2α in association with NB-UVB is considered therapeutically successful for vitiligo with non-significant difference between them. The onset of repigmentation was significantly earlier with PGE2 compared to PGF2α with subsequent significant higher patient satisfaction. Further studies are required to provide PGE2 and PGF2α in a formula suitable for direct intradermal injection.

## Data Availability

Data of the present study can be requested from the corresponding author.
